# Integrated, ultrafast all-optical polariton transistors with sub-wavelength grating microcavities

**DOI:** 10.1038/s41377-025-02050-2

**Published:** 2026-01-12

**Authors:** Pietro Tassan, Darius Urbonas, Bartos Chmielak, Jens Bolten, Thorsten Wahlbrink, Max C. Lemme, Michael Forster, Ullrich Scherf, Rainer F. Mahrt, Thilo Stöferle

**Affiliations:** 1 IBM Research Europe – Zurich, Rüschlikon, Switzerland; 2 Photonics Laboratory, ETH Zürich, Zürich, Switzerland Switzerland; 3 AMO GmbH, Aachen, Germany; 4 Chair of Electronic Devices, RWTH Aachen University, Aachen, Germany; 5 Macromolecular Chemistry Group and Wuppertal Center for Smart Materials & Systems (CM@S), Bergische Universität Wuppertal, Wuppertal, Germany

**Keywords:** Polaritons, Microresonators, Optoelectronic devices and components, Sub-wavelength optics, Integrated optics

## Abstract

All-optical logic has the potential to overcome the operation speed barrier that has persisted in electronic circuits for two decades. However, the development of scalable architectures has been prevented so far by the lack of materials with sufficiently strong nonlinear interactions needed to realize compact and efficient ultrafast all-optical switches with optical gain. Microcavities with embedded organic material in the strong light-matter interaction regime have recently enabled all-optical transistors operating at room temperature with picosecond switching times. However, the vertical cavity geometry, which is predominantly used in polaritonics, is not suitable for complex circuits with on-chip coupled transistors. Here, by leveraging state-of-the-art silicon photonics technology, we have achieved exciton-polariton condensation at ambient conditions in fully integrated high-index contrast sub-wavelength grating microcavities filled with a π-conjugated polymer as optically active material. We demonstrate ultrafast all-optical transistor action by coupling two resonators and utilizing seeded polariton condensation. With a device area as small as 2 × 2 µm^2^, we realize picosecond switching and amplification up to 60x, with extinction ratio up to 8:1. This compact ultrafast transistor device with in-plane integration is a key component for a scalable platform for all-optical logic circuits that could operate two orders of magnitude faster than electronic counterparts.

## Abstract (web summary)

Using integrated silicon high-contrast sub-wavelength gratings and organic cavity exciton-polaritons at room temperature, we realize all-optical transistor action with picosecond switching and amplification, as well as ultracompact footprint.

## Graphical abstract

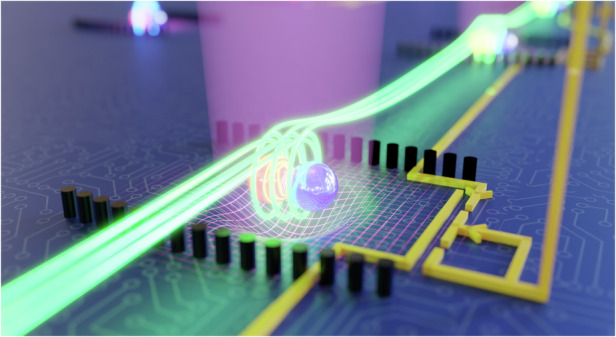

## Introduction

The clock speed of electronic circuits has been stagnant at a few gigahertz for almost two decades because of the breakdown of Dennard scaling^1^, which states that by shrinking the size of transistors they can operate faster while maintaining the same power consumption. All-optical logic with the ambition to supplement electrical circuits, particularly for high-speed signal processing and tasks that are not parallelizable^2^ and therefore cannot fully benefit from the ongoing density scaling of electronic transistors^3^, has been an active field of research for several decades^4–7^. On this journey, a large variety of device concepts has been explored, including optical bistability in semiconductor quantum wells and other materials^6^, semiconductor optical amplifiers^8^, resonators based on rings^9^, discs^10^ and photonic crystals^11^, plasmonics^12^, Mach-Zehnder interferometers^13^, excitons^14^ and single molecules^15^. While certain technologies enable operation at speeds that greatly exceed those of existing electronic circuits, achieving scalability for numerous interconnected all-optical gates continues to pose a significant challenge. This issue affects not only free-space architectures^16^ but is also attributed to the typically low intrinsic nonlinearities of materials and the necessity for precise sizing and matching of optical resonators throughout a chip^17^. To boost the nonlinearities, some recent approaches utilize the strong light-matter interaction regime, where an optically active material is embedded in a microcavity such that the coupling rate between optoelectronic excitations (correlated electron-hole pairs, excitons) in the material and photons in the cavity exceeds the loss rates in the material and the cavity^18^, effectively forming hybrid light-matter exciton-polariton quasi-particles. By using parametric scattering or resonant excitation, optical amplifiers^19^ as well as transistors^20^ and routers^21,22^ have been realized at cryogenic temperature, and more recently with lead halide perovskites at room temperature^23^.

With organics, in addition to the large exciton binding energy permitting exciton-polaritons at ambient conditions^24^, the polariton formation can be strongly enhanced by matching the detuning of the polariton state from the exciton reservoir to the energy of molecular vibrational modes^25^. At sufficiently high excitation density, nonlinear polariton condensation occurs^26,27^, resulting in coherent macroscopic occupation of a single polariton mode. Light inserted into the microcavity can seed and promote polariton condensation through bosonic stimulation, which can be harnessed for all-optical transistors and logic gates^28,29^ operating at sub-picosecond speed and controlled with tens or less signal photons^30^. However, a major downside common to these exciton-polariton devices is their vertical cavity design with distributed Bragg reflectors (DBR), resulting in a polariton condensate wavevector that is essentially perpendicular to the substrate. Such vertical configuration necessitates the use of external free-space optics to route emitted light between the individual transistors, resulting in significant propagation delays on the order of hundreds of picoseconds that ultimately prevent the realization of scalable ultrafast circuits. Alternative geometries without DBRs have employed waveguides with facets^31^ or microwires^32^ to demonstrate amplification and logic functionality with polaritons in ZnO, but a scaling path towards more complex circuits has been missing.

Sub-wavelength gratings built from materials with high refractive index contrast provide an alternative to DBRs^33,34^ and have already been used in a vertical microcavity structure to realize exciton-polariton lasing^35^. Fabricating pairs of high-contrast gratings (HCG) perpendicular to the chip substrate, compact 2 × 2 µm² Fabry-Perot like cavities can be created^36,37^ where the cavity mode is parallel to the substrate, enabling scalable in-plane architectures. Remarkably, compared to polariton condensation with metal^38,39^ and silicon^40^ metasurfaces, where the condensate extends over hundreds of micrometers, the effective modal area in HCG cavities is only about 1 µm^2^, enabling extraordinarily compact optical circuits with comparably high device densities, which is beneficial for lowering power consumption and high speed.

Here, we establish in-plane polariton condensation at room temperature by introducing compact integrated sub-wavelength grating cavities filled with an organic ladder-type polymer. With one HCG cavity generating the in-plane control signal that constitutes the input “seed” for a second “transistor” cavity, we demonstrate transistor action on a picosecond time scale. We observe up to 60x gain and a switching contrast exceeding 8:1 with an architecture that provides a scalability path through the planar on-chip nature of the signal routing and the compact device.

## Results

### On-chip polariton condensation

**Fig. 1:**
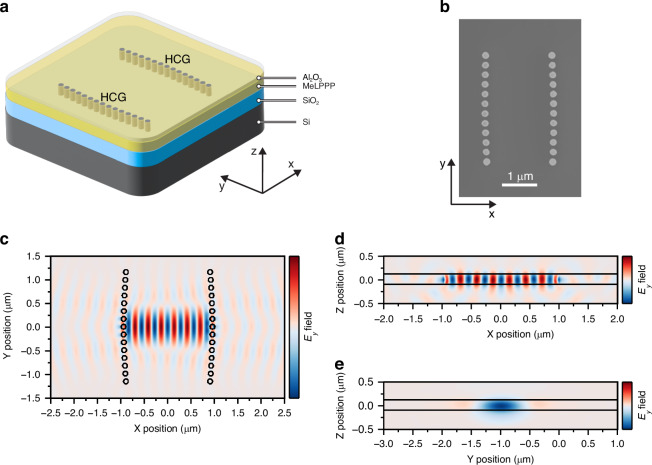
Integrated high contrast grating cavity. **a** Sketch of integrated high contrast grating (HCG) cavity embedded within the active polymer (MeLPPP) and encapsulation layer (Al_2_O_3_). **b** Scanning electron microscopy image (top view) of a fabricated device without polymer layer. **c**−**e** Different cross-sectional views of the electromagnetic field map of the resonant optical mode in the cavity, obtained using three-dimensional finite-difference time-domain (3D FDTD) simulations. The color scale shows the dominant y-component of the electric field (*E*_*y*_) of the optical mode. Overlaid in black are the outlines of the structure

As illustrated in Fig. 1a, the device comprises two HCG mirrors that provide lateral light confinement, and a polymer serving as both active material and guiding layer with vertical light confinement via total internal reflection, enabling in-plane propagation. The diameter and pitch of the nanostructured silicon pillars are designed to yield high in-plane reflectivity for transverse-electric light polarization at perpendicular incidence to the grating (see Fig. S1). The wide reflectivity maximum in spectrum and configuration space ensures broadband reflectivity and excellent robustness against fabrication errors. The HCG microcavity is fabricated on a silicon-on-insulator wafer using state-of-the-art silicon photonics technology and subsequent deposition of the ladder-type π-conjugated polymer (MeLPPP) and the encapsulation layer (see Methods and Fig. S2). Photonic simulations show the optical mode confinement within the device (Fig. 1c–e), resulting in a cavity quality factor of around *Q* ~ 420 for a wavelength of 490 nm, despite the strong optical absorption of the silicon constituting the HCGs. Notably, this *Q*-factor is sufficiently high to enable strong light-matter interaction and exciton-polariton condensation^26^, while still being low enough to ensure sub-picosecond polariton lifetimes and to relax the fabrication tolerance requirements for matching resonator wavelengths across the chip.

**Fig. 2:**
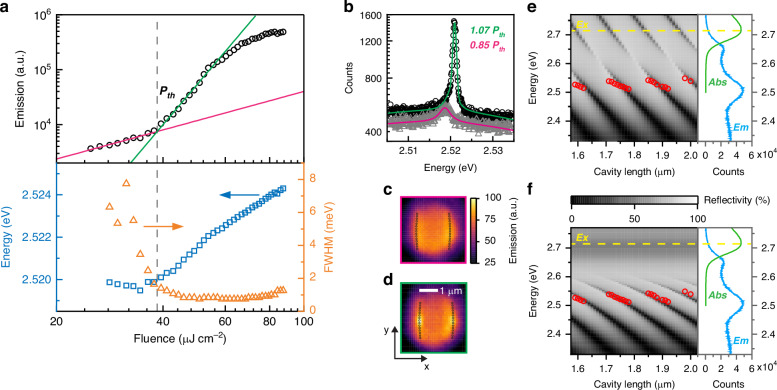
Strong light–matter interaction regime and polariton condensation. **a** The top panel shows the emission as a function of excitation fluence. This light-in light-out characteristic exhibits a nonlinear increase (power-law fit in green) above the threshold (*P*_th_ ~ 39 µJ cm^-2^, dashed line), after a linear emission (power-law fit in magenta), and saturation at higher fluences. The bottom panel displays the center energy of the emission peak (blue squares) and the width (orange triangles) as a function of excitation fluence, showing a sudden narrowing at the threshold and a continuous blue-shift above. **b** Emission spectra below threshold (gray data points) and above threshold (black data points), and their fitted spectra (magenta and green, respectively). **c** Real-space emission images of the cavity when pumped below and **d**, above threshold at the same fluences as the spectra in (**b**) were obtained. **e** Reflectivity (gray scale) obtained from a two-dimensional (2D) rigorous coupled wave analysis (RCWA) calculation as a function of cavity length in the weak coupling regime with refractive index *n* = 1.86 = const. and *k* = 0; the different longitudinal modes appear as dark stripes in the gray scale data. The experimentally measured energies (red circles) are extracted above threshold, where the nonlinear emission yields a sharper, more intense peak: these energies clearly are not matching the slope of the weak-coupling theory. The exciton energy is represented with a yellow dashed line at 2.714 eV. The right panel shows the absorption (green, Abs) and emission (blue, Em) spectra of a bare MeLPPP thin film without a cavity. **f** Simulated reflectivity (RCWA) as a function of cavity length for cavities in the strong-coupling regime (gray scale), where the full refractive index dispersion *n* = n(λ) and *k* = k(λ) is used, as obtained from ellipsometry of the polymer layer. The experimentally measured energies of the polariton condensates are overlaid as red circles. To account for the 2D nature of the simulation, which neglects the vertical guiding layer structure that results in a lower effective refractive index, the theoretical curves are shifted slightly (+0.07 µm in cavity length) to achieve a good match with the experiment. This offset dominates over the small energy blueshift (at most 5 meV) occurring above threshold, which is approximately rigid and thus negligible in comparison with the applied horizontal correction (~11 meV). The right panel shows the absorption (green) and emission (blue) spectra

We excite the structure from the top using a pulsed laser (see Methods). At low pump fluence, the intensity of the emission increases linearly (Fig. 2a, top panel), and a Lorentzian-shaped spectral peak with ~6 meV full-width at half-maximum (FWHM), consistent with the calculated cavity *Q*-factor, is observed along with a broad photoluminescence background. With increasing pump fluence, above threshold (*P*_th_ = 39 µJ cm^−2^), the emission peak acquires a Gaussian shape (Fig. 2b, inset and Fig. S3a) and undergoes a linewidth narrowing down to ~1 meV. Furthermore, a continuous blue-shift up to 5 meV from its original photon energy at a pump fluence of 90 µJ cm^−2^ is observed (Fig. 2a, bottom panel). To confirm that these observations are indeed signatures of polariton condensation, as already reported in vertical DBR cavities with the same active material and similar *Q*-factor^26^, we assess the presence of strong light-matter coupling in these structures by measuring a manifold of devices where the cavity length *L* is systematically varied, similar to the wedge-tuning in vertical DBR cavities^41,42^. Due to the in-plane architecture of our system, conventional white-light reflectivity or absorption measurements are not feasible, so instead, we probe the emission from the lower polariton branch: by plotting the resonance energies versus the cavity length *L*, we observe the characteristic bending of the lower polariton branch in the strong light–matter interaction regime, that is supported by comparing to simulations of the weakly-coupled and the strongly-coupled polymer-filled cavities (Fig. 2d, e) where the resonance energies change with a distinctly different slope with *L*. Fitting with a coupled-oscillators model (Fig. S4) yields a Rabi splitting of 2Ω = 327 ± 48 meV for an excitonic fraction of 50% at *L* = 1.7 µm. The large Rabi splitting and high excitonic fraction, compared to the values found in vertical DBR cavities where polariton condensation with MeLPPP was observed^26^ (2Ω ~ 120 meV and 20% exciton fraction), results from the significantly increased amount of polymer material in the cavity that is, however, partly offset by the reduced cavity finesse. Notably, for microcavities with MeLPPP, polariton condensation occurs only when the lower polariton branch is within ±20 meV the energy of the exciton reservoir (2.714 eV) minus the energy of the strong vibronic transition of the polymer^30^ (200 meV), where vibron-enhanced relaxation is prevalent^43^. The above-threshold emission of MeLPPP is reasonably stable, exhibiting only a few percent of photodegradation after several hours-long excitation (Fig. S3b). This could be potentially much further extended together with a further reduced threshold by changing the pump photon energy from 3.1 eV closer to the exciton resonance, resulting in less excess energy and higher absorption in the MeLPPP^30^.

Introducing a curvature to the HCGs allows us to engineer the modal structure of the microcavity. Gratings with a Gaussian shape, besides increasing the light confinement along the grating, lead to the discretization of the transversal modes (Fig. S5). The different transversal orders can be clearly identified from the spectrally filtered real-space emission patterns of the polariton condensates, which show the parasitic vertically scattered light of the in-plane mode by the HCGs. Thus, by engineering the cavity length and curvature, we can ensure single mode polariton condensates with well-defined polarization (Fig. S6), an important feature for controlled coupling of multiple HCG resonators.

For scaling to circuits with many devices, it is crucial that the resonance frequency of the fabricated HCG cavities is well controlled, and deviations stay within the linewidth of the resonators. The fact that the resonance energies of more than three hundred HCG cavities line up almost perfectly in the study of the mode structure (Fig. S5), suggests good homogeneity and precision of the fabrication processes. Moreover, we studied the statistical distribution of polariton condensate emission energies of >30 nominally identical fabricated cavities (Fig. S7), resulting in a standard deviation of <0.5 meV, much below the cavity linewidth (FWHM ~ 6 meV). Hence, even accounting for blue-shift of the condensate emission, this fulfils a pivotal condition for device cascadability where the output of one cavity serves as input of another one.

To realize ultrafast devices, it is important to assess the dynamics of the polariton condensation. By exciting a single HCG cavity above threshold (~1.2 *P*_th_) with a femtoseconds-pulsed laser and interfering the real-space images by means of a Michelson interferometer with a retroreflector mounted in one arm, we measured temporal and spatial coherence (Fig. S8). The coherence time of the condensate, which is directly related to its lifetime, shows a FWHM ~270 fs, providing an estimate for the ultrafast pulse duration generated as HCG cavity output. Furthermore, we performed an experiment with a bi-injection scheme where we excited a single HCG cavity with two subsequent pulses of the same fluence and same duration (~150 fs), while precisely tuning the time delay between them. When the fluence of a single pulse is below threshold, the polariton condensation process with its concomitant increase in emission is triggered only when the second pulse arrives within about 1 ps (Fig. S9), which we therefore attribute to an intrinsic relaxation time, consistent with modeling and experimental results with MeLPPP polaritons in vertical cavities^29,44^. For larger time delays, a much longer lifetime component around 22 ps is observed that corresponds to the lifetime of the excitons. Hence, from the time scale of the initial fast drop, the maximum repetition rate of the devices can be inferred, and the height of the long tail versus the initial peak gives a limit for the extinction.

### In-plane transistor action

**Fig. 3:**
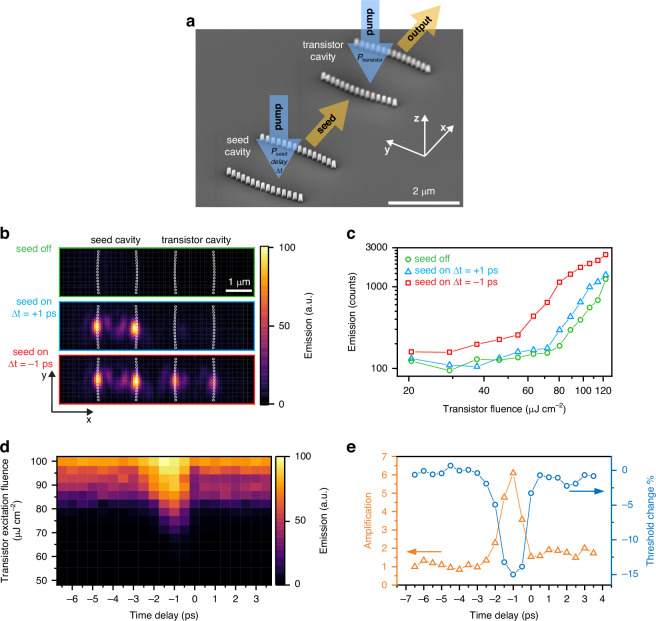
Ultrafast transistor action. **a** Illustration where a scanning electron microscopy image of the seed and the transistor cavity without polymer layer is overlayed with arrows for the excitation beams and the output of the seed cavity flowing to the transistor cavity. **b** Real-space images of the emission with excitation of the transistor cavity at *P*_transistor_ = 0.8 *P*_th_. In the top green panel, the seed cavity is not excited. In the middle blue panel, it is excited with *P*_seed_ = 1.2 *P*_th_ at a time delay of Δ*t* = +1 ps after the excitation of the transistor cavity. In the bottom red panel, *P*_seed_ = 1.2 *P*_th_ at Δ*t* = -1 ps before the excitation of the transistor cavity. **c** Integrated intensity of the emitted spectrum collected from the output mirror of the transistor cavity shown as a function of transistor excitation fluence when the transistor cavity is excited at *P*_transistor_ = 0.8 *P*_th_ and the seed cavity is not pumped (green), is excited with *P*_seed_ = 1.2 *P*_th_ at a time delay of Δ*t* = +1 ps (blue) and with *P*_seed_ = 1.2 *P*_th_ at a time delay of Δ*t* = -1 ps (red). **d** Map of the transistor output emission intensity versus transistor excitation fluence and time delay between seed and transistor excitation for *P*_seed_ = 1.5 *P*_th_. **e** Threshold reduction of the transistor cavity (blue) and amplification of the seed (orange) as a function of seed–transistor excitation time delay

Next, we study a configuration comprising two cavities with curved HCG mirrors, having the same resonance energy and being spatially separated by a 3 µm gap. We excite independently each cavity with an ultrafast pulse of controlled fluence and a variable time delay Δ*t* between the two pulses (Fig. 3a). In this scheme, we use the output photons of one cavity, representing the control gate or “seed”, as input for the other cavity, representing the “transistor”. The polymer layer, that covers both structures as well as the space between them, serves as a waveguide with vertical confinement realized through total internal reflection due to the polymer’s higher refractive index than the SiO_2_ layer and air cladding below and above, respectively. This vertical confinement, combined with low lateral divergence, allows the emission from the seed cavity to couple into the same mode of the transistor cavity after only few femtoseconds propagation due to the short distance between them. We estimate a coupling efficiency of ~84%, obtained from the ratio of the integrated intensity of parasitically scattered light from the transistor output mirror and the transistor input mirror when the seed cavity is pumped but the transistor cavity is not excited.

Real-space images of the emission (Fig. 3b) indicate that the transistor can still achieve polariton condensation when excited below threshold (*P*_transistor_ = 0.8 *P*_th_), but only if the seed cavity is excited above its condensation threshold (*P*_seed_ = 1.2 *P*_th_) slightly before the transistor cavity with a negative seed–transistor pump pulse time delay around Δ*t* = −1 ps. We record the output intensity of the transistor cavity by collecting the emission spectrum only from the transistor HCG mirror that is on the far side from the seed, i.e. the transistor output. When we plot the emission intensity versus the excitation fluence (Fig. 3c), we find that the condensation threshold is reduced only when the gating signal from the seed is present and arrives with the aforementioned timing, but not when the seed cavity is excited later (Δ*t* = +1 ps).

To map the temporal dynamics, we vary both the transistor excitation fluence and the time delay Δ*t* between the excitation pulses for the seed and the transistor cavity, for a fixed seed excitation fluence of *P*_seed_ = 1.5 *P*_th_ (Fig. 3d). Comparing the emission intensities, we observe that signal amplification (Fig. 3e) in the transistor only occurs when the seed cavity is excited ~1 ps before the transistor cavity. This optimal timing condition reflects the intrinsic relaxation dynamics within the seed cavity, as demonstrated by the two-pulse experiment in a single HCG cavity, and should not be interpreted as the time needed for photons to propagate between cavities, which is only tens of femtoseconds. Furthermore, we observe a significant threshold reduction of the transistor cavity (Fig. 3e), which for this seed fluence is about 15%, but can reach >30% with stronger seed (*P*_seed_ = 2 *P*_th_).

As both seed and transistor cavities are nominally identical, they can switch roles. When we detect the light on the HCG mirror of the seed cavity on the far side of the transistor cavity, we observe the mirrored temporal dynamics compared to Fig. 3d (Fig. S10), with a higher background due to the above-threshold excitation of the seed cavity (*P*_seed_ = 1.2 *P*_th_). This demonstrates that the excitation pulse sequence determines the directional flow of the signal on the chip, suggesting that scalable circuits comprising multiple successive transistors with signal routing free from complications arising from back-action can be developed by utilizing customized excitation sequences. Furthermore, in contrast to vertical geometries^28,29^, which need to reroute the off-chip transistor output back onto the chip, thereby incurring hundreds of picoseconds latency, the propagation time of the photons between different integrated cavities here is on the order of tens of femtoseconds.

### Transistor metrics

**Fig. 4:**
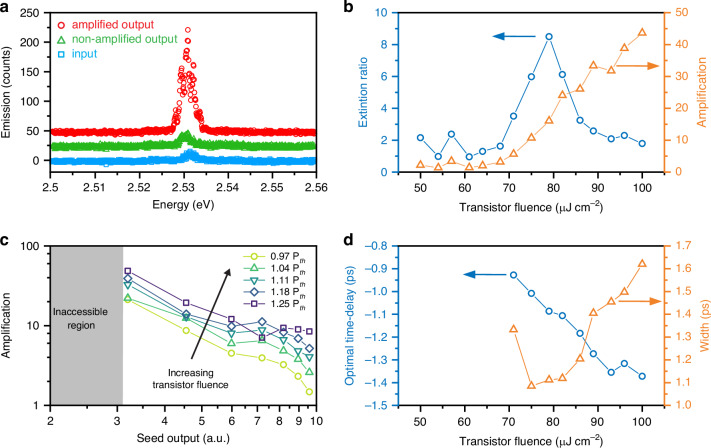
Spectra and transistor performance. **a** Comparison between the input signal (blue), the transistor output spectrum without amplification (green) and the amplified signal (red), offset vertically by 25 counts each. The spectra have been collected at the transistor output mirror when the seed is excited with *P*_seed_ = 1.2 *P*_th_ and the transistor with *P*_transistor_ = 0.6 *P*_th_ (blue) and the transistor with *P*_transistor_ = 1.4 *P*_th_ at Δ*t* = +1 ps (seed pumped after transistor, green) and Δ*t* = -1 ps (seed pumped before transistor, red), respectively. **b** Extinction ratio (blue) and amplification (orange) of the transistor versus transistor excitation fluence. **c** The transistor amplification varies as a function of the intensity of the input signal, i.e. the seed cavity output which is related to the seed excitation fluence, and with the transistor excitation fluence. In the inaccessible region, the seed cavity excitation is too weak to induce polariton condensation in the seed cavity thereby preventing generation of a short input pulse for the transistor. **d** Optimal time delay (blue) and width of the amplification time-window (red) as function of the transistor excitation fluence

We extract key transistor metrics from the emission spectra and their temporal dynamics. When comparing the emission spectrum at the transistor output without and with applying the excitation to the transistor cavity (Fig. 4a), we observe that the amplified peak faithfully reproduces the input from the seed, only negligibly changing its energy and spectral shape. Likewise, comparing spectra from the transistor output without and with seed excitation shows that the output spectrum remains essentially unchanged apart from the intensity change (Fig. 4a), allowing the extraction of the extinction ratio (on/off) of the transistor. The absence of significant spectral distortion is a fundamental requirement for the cascadability of the devices. We plot the transistor metrics as a function of transistor excitation fluence (Fig. 4b), showing that for a given seed excitation fluence (*P*_seed_ = 1.5 *P*_th_) the amplification increases with the transistor excitation up to a value of 43, and the extinction reaches a ratio of 8:1 near the threshold. Varying the seed excitation fluence, and thereby the intensity of the gating input to the transistor, we find that the signal amplification increases for smaller input signals (Fig. 4c), reaching 60 for the smallest inputs that we could produce without losing polariton condensation in the seed cavity. At this condition, where the seed pumping fluence is just above the seed cavity condensation threshold, also the switching energy is minimal, which is around ~10 fJ (for details see “Definition and Extraction of Key Performance Metrics” in Supplementary Information).

Furthermore, to assess the temporal robustness of the transistor amplification, we investigate how the amplification dynamics change with transistor excitation fluence (Fig. 4d). We find that the time delay between the seed and the transistor excitation where the maximum amplification is achieved slightly shifts from −0.9 ps to −1.4 ps with increasing transistor excitation. This can be understood considering that for stronger transistor excitation, the spontaneous polariton condensation is generally more dominant, and therefore the seed light must be injected early enough into the cavity to stimulate scattering into the seeded mode and suppress spontaneous condensation.

## Discussion

An aspect that could become important with future, highly-dense circuits is crosstalk. In this respect, the lateral confinement provided by the curved HCG mirrors and the resulting discretization of transverse modes serve to suppress inter-cavity crosstalk as higher-order modes, where present, would be filtered out due to their poor spatial overlap and mismatched resonance with neighboring cavities. Furthermore, the tightly focused laser excitation ensures spatially localized pumping, effectively avoiding unwanted excitation of adjacent cavities. Finally, the pump sequence with the required seed-transistor delay intrinsically promotes unidirectional signal propagation between cavities, mitigating back-action and ensuring directional control even in multi-device configurations.

In conclusion, we realized optically-pumped room-temperature polariton condensates that, in contrast to previous non-scalable architectures, feature a single micrometer-sized condensate mode that is parallel to the chip surface, enabling integrated planar circuits. By cascading two ultra-compact resonators in-plane, we demonstrated all-optical ultrafast transistor action with femtojoule-level input signals and signal amplification of up to a factor of 60 that can enable large fan-out and provide scalability beyond this basic building block (for benchmarks see Table S1 in Supplementary Information). Harnessing silicon photonics nanofabrication technology and exploiting the outstanding engineerability of the HCG cavities^45^, we expect our approach to be scalable to combine several transistors to logic gates^28^ and more complex logic circuits. Using waveguides with either HCGs^46^ or silicon nitride co-integration^47,48^ would allow routing the signals between distant transistors as well as the excitation pulses for the polariton condensates, opening the door towards a platform for all-optical logic circuits that may operate significantly faster than electronic ones.

## Materials and methods

### Device fabrication

We have fabricated the HCGs on silicon-on-insulator (SOI) wafers with 220 nm top-Si thickness and 3 µm buried oxide using high-resolution silicon photonics technology, where electron beam lithography (Raith EBPG 5200) exposes hydrogen silsesquioxane (HSQ) resist with 200 nm layer thickness. After development with tetramethylammonium hydroxide (TMAH), the structures were transferred by Cl-based reactive ion etching (RIE) into the top-Si layer. This results in a grating post diameter of 100 nm and a pitch of 165 nm. Methyl-substituted ladder-type poly(p-phenylene) (MeLPPP; *Mn* = 31,500 (number averaged molecular weight), *Mw* = 79,000 (weight averaged molecular weight)) was synthesized as described elsewhere^49^. MeLPPP is dissolved in toluene and spin-coated on the fabricated HCG structures, resulting in approximately 220 nm layer thickness, verified by profilometry and ellipsometry. A 20 nm-thick encapsulation layer of Al_2_O_3_ is evaporated on top for protection against photodegradation.

### Photonic simulations

For the computation of the HCG reflectivity and the spectra of the empty cavity and filled with polymer, we perform rigorous coupled wave analysis (RCWA) using a freely available software package^50^. For the three-dimensional finite-difference time-domain (3D FDTD) simulations, we use the commercial software ANSYS Lumerical. For both calculations, we use the complex refractive index obtained from variable-angle spectroscopic ellipsometry measurements (Woollam VASE) of the polymer layer on a test silicon wafer.

### Optical characterization

We mount the 20 mm × 20 mm chips, each containing hundreds of HCG cavities, on an *XYZ* nanopositioning stage in ambient conditions. Ultrafast excitation light pulses of 400 nm wavelength, 150 fs pulse duration and 1 kHz repetition rate are generated from a frequency-doubled regenerative amplifier that is seeded by a mode-locked Ti:sapphire laser. For the cavity detuning experiments with single HCG cavities, we insert the pulsed light into a photonic crystal fiber that stretches the pulse duration to >5 ps and provides a near-Gaussian beam at its output. The light is focused through a microscope objective (Mitutoyo Plan Apo 100X, NA = 0.7) to an approximately Gaussian spot size of ~2 µm e^−2^ diameter on the sample. The beam is incident along the optical axis of the objective (i.e., normal to the chip surface) (Fig. S11).

For single-cavity condensation dynamics and transistor operation, the laser beam is split into two paths with independent delay lines and variable power control. Both beams are recombined collinearly and coupled into the same objective. The spatial position of the beam on the chip is aligned manually via imaging of the beams and refined via nanopositioning stages to ensure targeted illumination of either single cavities or cavity pairs, depending on the experiment.

Time delay between the two excitation pulses is tuned using a motorized delay stage with a resolution of Δ*x* = 100 nm, corresponding to a temporal resolution Δ*t* = 0.67 fs (derived from Δ*t* = 2Δ*x*/*c*, with *c* being the speed of light). Emitted light is collected through the same objective and separated from excitation via a dichroic mirror and long-pass filters. We detect the emission with a 50:50 beam splitter simultaneously with a camera and a spectrometer equipped with a liquid-nitrogen-cooled CCD. For spectral measurements, the 0.5 m-long spectrometer uses an 1800 lines per mm grating, yielding a dispersion of ~0.05 nm per pixel (at 490 nm). The spectrometer receives light via a multimode fiber with either 10 μm or 100 μm core diameter, resulting in detection spots of ~1 μm and ~10 μm, respectively, on the sample. The spatial resolution of our optical system, determined by fitting the point spread function (PSF) of scattered light from a single point emitter, yields a FWHM of ~477 nm, which corresponds to an effective Rayleigh resolution of ~649 nm, close to the theoretical diffraction limited resolution of ~430 nm (for λ = 492 nm and NA = 0.7).

## Supplementary information

Supplementary Information

## Data Availability

Data supporting the findings of this study are available from the corresponding authors upon reasonable request.

## References

[1] Dennard, R. H. et al. Design of ion-implanted MOSFET’s with very small physical dimensions. *IEEE J. Solid-State Circuits* **9**, 256–268 (1974).

[2] Greenlaw, R., Ruzzo, W. L. & Hoover, J. *A Compendium of Problems Complete for P (Preliminary)* (University of Washington, 1991).

[3] Datta, S., Chakraborty, W. & Radosavljevic, M. Toward attojoule switching energy in logic transistors. *Science* **378**, 733–740 (2022). 36395210 10.1126/science.ade7656

[4] Miller, D. A. B. Are optical transistors the logical next step? *Nat. Photonics* **4**, 3–5 (2010).

[5] Caulfield, H. J. Perspectives in optical computing. *Computer* **31**, 22–25 (1998).

[6] Gibbs, H. M. *Optical Bistability: Controlling Light with Light* (Orlando: Academic Press, 1985).

[7] Haus, H. A. & Whitaker, N. A. All-optical logic in optical waveguides. *Philos. Trans. R. Soc. Lond. Ser. A, Math. Phys. Sci.* **313**, 311–319 (1984).

[8] Stubkjaer, K. E. Semiconductor optical amplifier-based all-optical gates for high-speed optical processing. *IEEE J. Sel. Top. Quant. Electron.* **6**, 1428–1435 (2000).

[9] Almeida, V. R. et al. All-optical control of light on a silicon chip. *Nature* **431**, 1081–1084 (2004). 15510144 10.1038/nature02921

[10] Liu, L. et al. An ultra-small, low-power, all-optical flip-flop memory on a silicon chip. *Nat. Photonics* **4**, 182–187 (2010).

[11] Nozaki, K. et al. Sub-femtojoule all-optical switching using a photonic-crystal nanocavity. *Nat. Photonics* **4**, 477–483 (2010).

[12] Fu, Y. L. et al. All-optical logic gates based on nanoscale plasmonic slot waveguides. *Nano Lett.* **12**, 5784–5790 (2012). 23116455 10.1021/nl303095s

[13] Tang, X. F. et al. A reconfigurable optical logic gate with up to 25 logic functions based on polarization modulation with direct detection. *IEEE Photonics J.* **9**, 1–11 (2017).

[14] Kuznetsova, Y. Y. et al. All-optical excitonic transistor. *Opt. Lett.* **35**, 1587–1589 (2010). 20479817 10.1364/OL.35.001587

[15] Hwang, J. et al. A single-molecule optical transistor. *Nature* **460**, 76–80 (2009). 19571881 10.1038/nature08134

[16] McCormick, F. B. et al. Six-stage digital free-space optical switching network using symmetric self-electro-optic-effect devices. *Appl. Opt.* **32**, 5153–5171 (1993). 20856323 10.1364/AO.32.005153

[17] Fushimi, A. & Tanabe, T. All-optical logic gate operating with single wavelength. *Opt. Express* **22**, 4466–4479 (2014). 24663768 10.1364/OE.22.004466

[18] Kavokin, A. et al. *Microcavities* 2nd edn (Oxford: Oxford University Press, 2017).

[19] Savvidis, P. G. et al. Angle-resonant stimulated polariton amplifier. *Phys. Rev. Lett.* **84**, 1547–1550 (2000). 11017564 10.1103/PhysRevLett.84.1547

[20] Ballarini, D. et al. All-optical polariton transistor. *Nat. Commun.* **4**, 1778 (2013). 23653190 10.1038/ncomms2734

[21] Marsault, F. et al. Realization of an all optical exciton-polariton router. *Appl. Phys. Lett.* **107**, 201115 (2015).

[22] Schmutzler, J. et al. All-optical flow control of a polariton condensate using nonresonant excitation. *Phys. Rev. B.* **91**, 195308 (2015).

[23] Feng, J. G. et al. All-optical switching based on interacting exciton polaritons in self-assembled perovskite microwires. *Sci. Adv.* **7**, eabj6627 (2021). 34757800 10.1126/sciadv.abj6627 PMC8580323

[24] Lidzey, D. G. et al. Strong exciton–photon coupling in an organic semiconductor microcavity. *Nature* **395**, 53–55 (1998).

[25] Coles, D. M. et al. Vibrationally assisted polariton-relaxation processes in strongly coupled organic-semiconductor microcavities. *Adv. Funct. Mater.* **21**, 3691–3696 (2011).

[26] Plumhof, J. D. et al. Room-temperature Bose–Einstein condensation of cavity exciton–polaritons in a polymer. *Nat. Mater.* **13**, 247–252 (2014). 24317189 10.1038/nmat3825

[27] Daskalakis, K. S. et al. Nonlinear interactions in an organic polariton condensate. *Nat. Mater.* **13**, 271–278 (2014). 24509602 10.1038/nmat3874

[28] Zasedatelev, A. V. et al. A room-temperature organic polariton transistor. *Nat. Photonics* **13**, 378–383 (2019).

[29] Sannikov, D. A. et al. Room temperature, cascadable, all-optical polariton universal gates. *Nat. Commun.* **15**, 5362 (2024). 38918407 10.1038/s41467-024-49690-3 PMC11199649

[30] Zasedatelev, A. V. et al. Single-photon nonlinearity at room temperature. *Nature* **597**, 493–497 (2021). 34552252 10.1038/s41586-021-03866-9

[31] Jamadi, O. et al. Edge-emitting polariton laser and amplifier based on a ZnO waveguide. *Light Sci. Appl.* **7**, 82 (2018). 30393535 10.1038/s41377-018-0084-z PMC6207564

[32] Li, H. et al. All-optical temporal logic gates in localized exciton polaritons. *Nat. Photonics* **18**, 864–869 (2024).

[33] Mateus, C. F. R. et al. Ultrabroadband mirror using low-index cladded subwavelength grating. *IEEE Photonics Technol. Lett.* **16**, 518–520 (2004).

[34] Chang-Hasnain, C. J. & Yang, W. J. High-contrast gratings for integrated optoelectronics. *Adv. Opt. Photonics* **4**, 379–440 (2012).

[35] Zhang, B. et al. Zero-dimensional polariton laser in a subwavelength grating-based vertical microcavity. *Light Sci. Appl.* **3**, e135 (2014).

[36] Stöferle, T. et al. Ultracompact silicon/polymer laser with an absorption-insensitive nanophotonic resonator. *Nano Lett.* **10**, 3675–3678 (2010). 20722400 10.1021/nl102149y

[37] Urbonas, D. *Tunable Coupled Microcavities for Enhanced Light-Matter Interaction* (ETH Zurich, Zurich, 2019).

[38] Ramezani, M. et al. Plasmon-exciton-polariton lasing. *Optica* **4**, 31–37 (2017).

[39] Hakala, T. K. et al. Bose–Einstein condensation in a plasmonic lattice. *Nat. Phys.* **14**, 739–744 (2018).

[40] Castellanos, G. W. et al. Non-equilibrium Bose–Einstein condensation of exciton-polaritons in silicon metasurfaces. *Adv. Optical Mater.* **11**, 2202305 (2023).

[41] Stanley, R. P. et al. Cavity-polariton photoluminescence in semiconductor microcavities: experimental evidence. *Phys. Rev. B.* **53**, 10995–11007 (1996). 10.1103/physrevb.53.10995 9982672

[42] Wenus, J. et al. Tuning the exciton-photon coupling in a strongly coupled organic microcavity containing an optical wedge. *Appl. Phys. Lett.* **85**, 5848–5850 (2004).

[43] Mazza, L. et al. Microscopic theory of polariton lasing via vibronically assisted scattering. *Phys. Rev. B.* **88**, 075321 (2013).

[44] Misko, M. et al. Temporal bandwidth of consecutive polariton condensation. *Phys. Rev. B.* **111**, L161403 (2025).

[45] Fischbach, J. D. et al. A framework to compute resonances arising from multiple scattering. *Adv. Theory Simul.* **8**, 2400989 (2025).

[46] Urbonas, D., Mahrt, R. F. & Stöferle, T. Low-loss optical waveguides made with a high-loss material. *Light Sci. Appl.* **10**, 15 (2021). 33436556 10.1038/s41377-020-00454-w PMC7804948

[47] Cegielski, P. J. et al. Monolithically integrated perovskite semiconductor lasers on silicon photonic chips by scalable top-down fabrication. *Nano Lett.* **18**, 6915–6923 (2018). 30278610 10.1021/acs.nanolett.8b02811

[48] Chauhan, N. et al. Ultra-low loss visible light waveguides for integrated atomic, molecular, and quantum photonics. *Opt. Express* **30**, 6960–6969 (2022). 35299469 10.1364/OE.448938

[49] Scherf, U., Bohnen, A. & Müllen, K. Polyarylenes and poly(arylenevinylene)s, 9^+^ the oxidized states of a (1,4-phenylene) ladder polymer. *Die Makromol. Chem.* **193**, 1127–1133 (1992).

[50] Liu, V. & Fan, S. H. S4: a free electromagnetic solver for layered periodic structures. *Comput. Phys. Commun.* **183**, 2233–2244 (2012).

