# Supplementary material for: Integrated, ultrafast all-optical polariton transistors with sub-wavelength grating microcavities
Source: Light Sci Appl. 2026 Jan 12;15:65. doi: 10.1038/s41377-025-02050-2 (PMC12791137; doi:10.1038/s41377-025-02050-2)
Supplement: Supplementary file 1 — Supplementary Information [file 41377_2025_2050_MOESM1_ESM.pdf]

SUPPLEMENTARY INFORMATION FOR

Integrated, ultrafast all-optical polariton transistors with sub-wavelength grating microcavities

Pietro Tassan[1,2,+], Darius Urbonas[1,+,#,*], Bartos Chmielak[3], Jens Bolten[3], Thorsten Wahlbrink[3], Max C. Lemme[3,4], Michael Forster[5], Ullrich Scherf[5], Rainer F. Mahrt[1], and Thilo Stöferle[1,#,*]

[1] IBM Research Europe – Zurich, Rüschlikon, Switzerland

[2] Photonics Laboratory, ETH Zürich, Zürich, Switzerland, Switzerland

[3] AMO GmbH, Aachen, Germany

[4] Chair of Electronic Devices, RWTH Aachen University, Aachen, Germany

[5] Macromolecular Chemistry Group and Wuppertal Center for Smart Materials & Systems (CM@S), Bergische Universität Wuppertal, Wuppertal, Germany

[+] These authors contributed equally to this work.

[#] These authors have jointly supervised this work.

[*] Corresponding authors: dar@zurich.ibm.com, tof@zurich.ibm.com

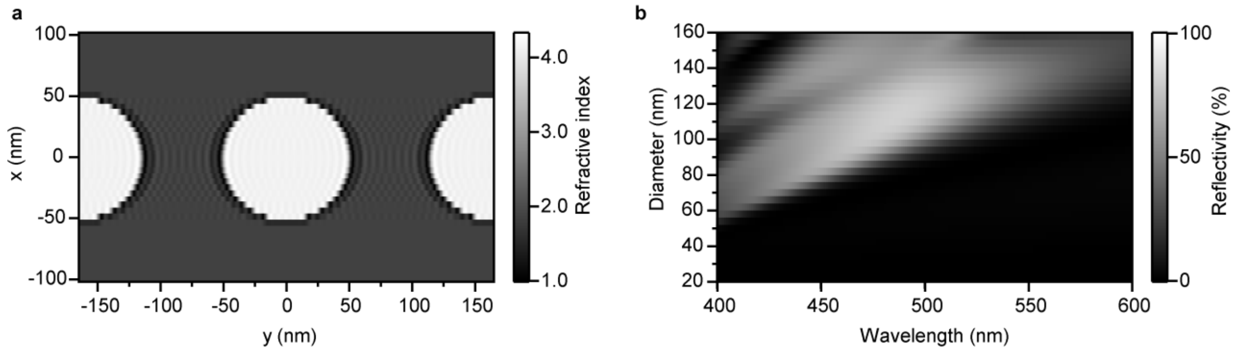

Fig. S1 | High contrast grating mirrors. a, Map showing the spatial distribution of the refractive index along a silicon–MeLPPP HCG as used with the RCWA simulation. **b**, Reflectivity map of a HCG mirror as function of wavelength and diameter of the silicon pillars constituting the grating.

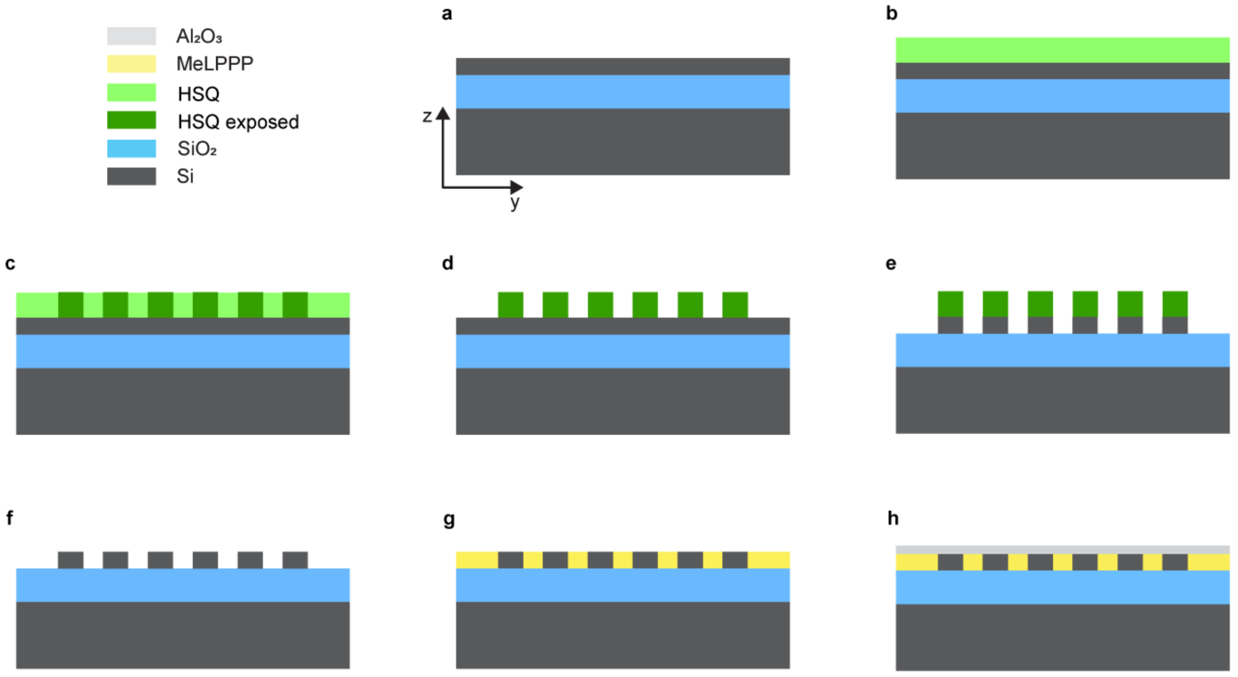

Fig. S2 | Fabrication process of high contrast grating microcavities. a, SOI wafer with top silicon layer of 220 nm and 3 µm thick silica layer. **b**, Spin-coating deposition of HSQ resist. **c**, Exposure and patterning of HSQ with electron beam lithography. **d**, Development of HSQ. **e**, Etching of uncovered silicon. **f**, Removal of residual HSQ. **g**, Spin-coating deposition of MeLPPP. **h**, Encapsulation with alumina through electron beam deposition.

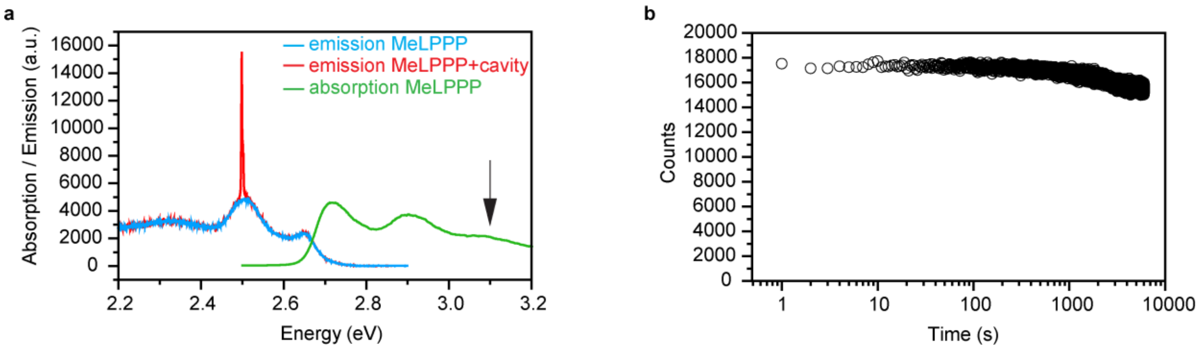

Fig. S3 | MeLPPP spectrum and photostability. a, Photoluminescence spectra of MeLPPP measured by exciting at 3.1 eV (black arrow) with ~1.5 P_{th} on top of a HCG cavity (red) and outside of a HCG cavity (blue) and absorption spectrum (green). **b**, Spectrally integrated photoluminescence intensity measured outside of the HCG cavity as a function of time, excited at a fluence of ~1.5 P_{th} using 150 fs pulses with 1 kHz repetition rate.

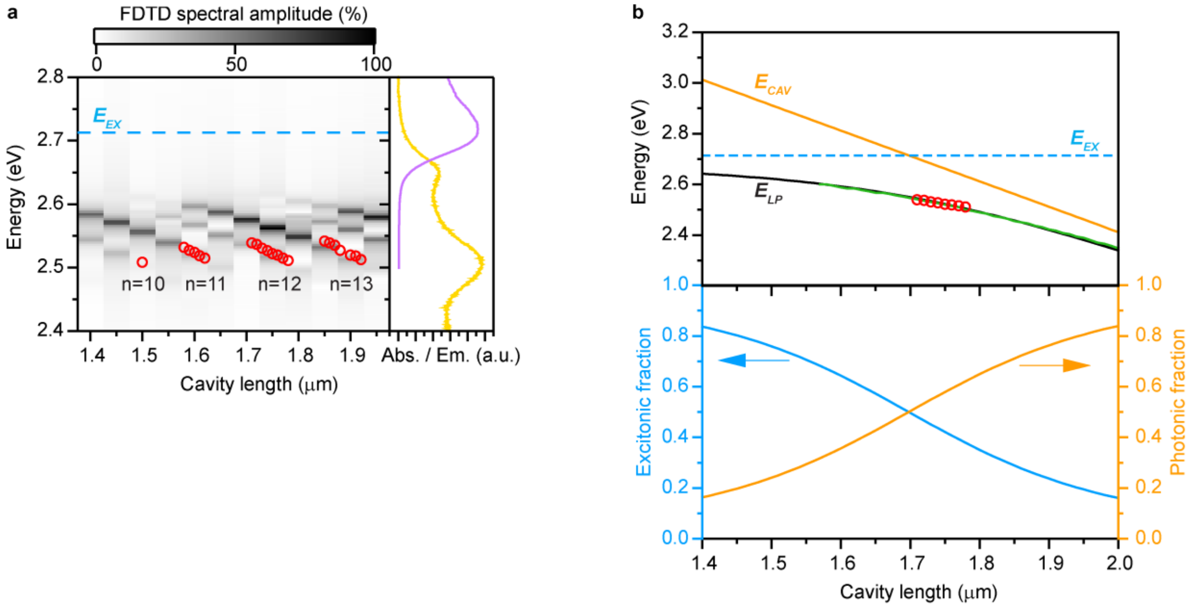

Fig. S4 | Simulations and coupled-oscillators model. a, Cavity detuning plot (energy versus cavity length) of experimentally measured polariton condensation energies (red circles) and simulated spectral resonances (grey scale), designated by their longitudinal order *n*, as obtained from 3D FDTD calculations of HCG cavities in the strong light-matter interaction regime. The ab-initio simulations use the as-designed geometrical parameters and the measured refractive index dispersion from MeLPPP without any adjustments made to match to the experimental resonances. The exciton energy is represented by the dashed blue line. The right panel shows the absorption (purple) and emission (yellow) spectra of MeLPPP. **b**, Top panel shows the experimentally measured resonance energies of the longitudinal mode with n = 12 (red circles) and a fit with a coupled-oscillators model showing the lower polariton branch (black). Cavity resonance energies displaying the strong light-matter interaction regime (green) and the weak-coupling regime (orange) have been extracted from RCWA simulations versus cavity length. The bottom panel shows the exciton (blue) and photon (orange) fraction, i.e. Hopfield coefficients, of the lower polariton branch versus the cavity length for this mode.

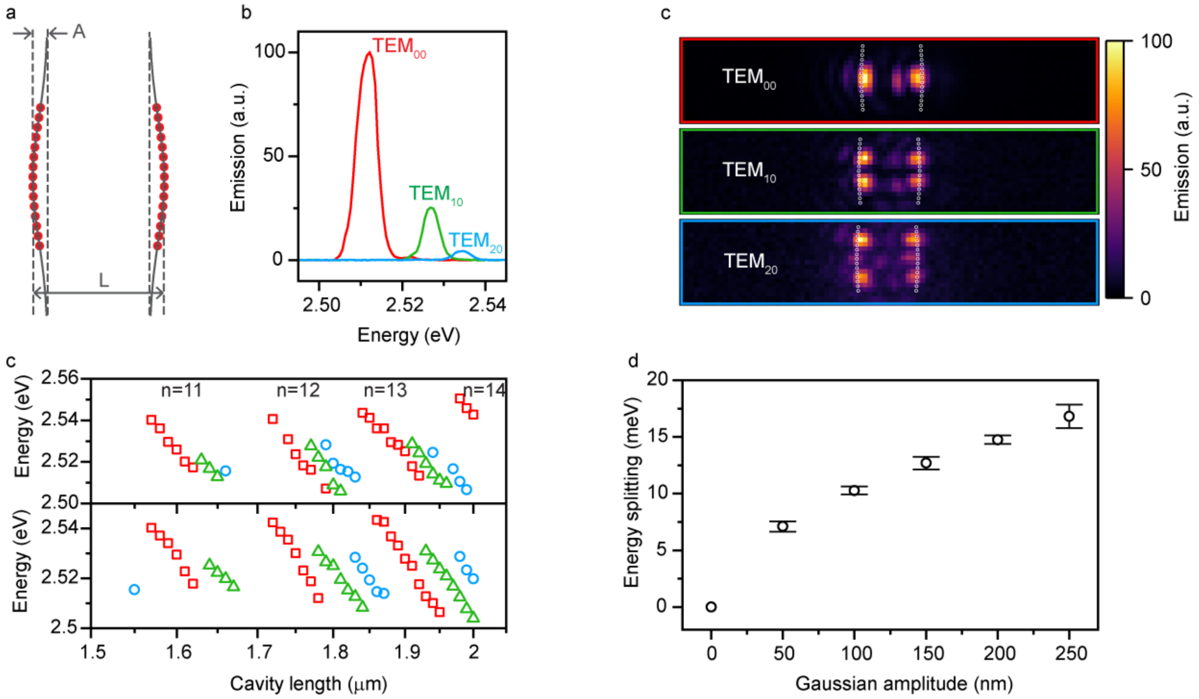

Fig. S5 | Engineering the high contrast grating cavity modes. a, Scheme of a HCG cavity, highlighting the Gaussian curvature of the mirrors. **b**, Three energy-filtered emission spectra from a HCG cavity with cavity length L = 1.92 μm and curvature described by a Gaussian amplitude of A = 100 nm and a FWHM of 2.36 μm. The spectra relate to modes of different transversal order: TEM_{00} (red), TEM_{10} (green), TEM_{20} (blue). **c**, Real-space images displaying the energy-filtered emission for the three modes shown in **a** with their characteristic scattering pattern on the HCG mirrors that reflects the modal profile in the cavity. **d**, Energy of cavity resonances as a function of cavity length for Gaussian-shaped cavities with A = 100 nm (top panel) and A = 250 nm (bottom panel). Different transversal orders are highlighted by different colors and symbols with TEM_{00} (red), TEM_{10} (green), TEM_{20} (blue). **e**, Energy splitting between TEM_{00} and TEM_{10} as a function of Gaussian amplitude A. For the flat cavities (A = 0 nm), there is no splitting as there are no discrete transversal orders. Vertical bars indicate the standard deviations of the energy splitting resulting from measurements on different devices having different cavity lengths.

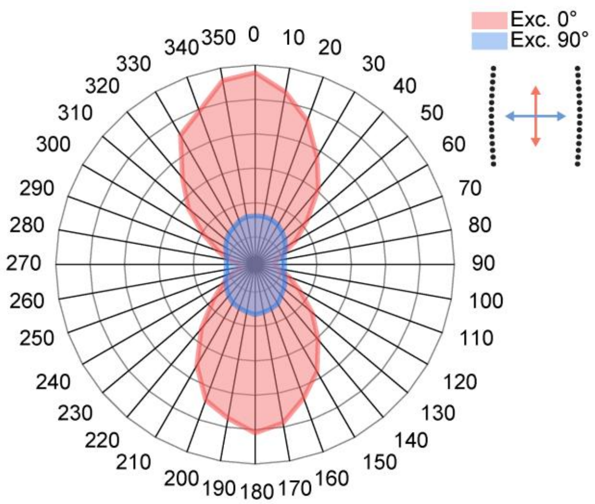

Fig. S6 | Polarization of the emission. Emission intensity of the HCG cavity is shown as a function of angle of a polarizer that is placed in the detection path. When the excitation polarization is aligned parallel to the HCG gratings (red), polariton condensation occurs in the cavity with a mode polarization that is parallel to the HCG gratings. When excited with the same fluence but with orthogonal polarization (blue), no polarition condensation takes place, and the emission shows an almost angularly invariant pattern, typical for the unpolarized photoluminescence from a MeLPPP film, presumably slightly modified by the scattering from the HCG gratings.

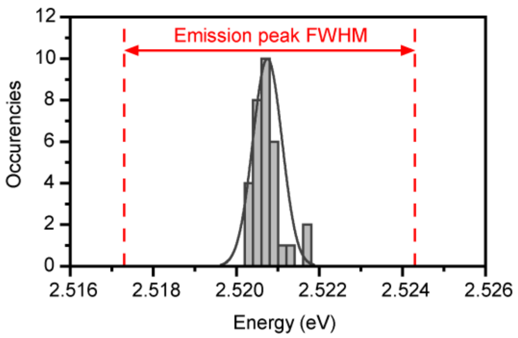

Fig. S7 | Statistics on high contrast grating cavities. The distribution of the measured energy of the center of the condensation peaks of 32 HCG microcavities, which are identical by design (L = 1.91 μm), is represented with the gray histogram with bin size of 0.2 meV and is fitted with a normal distribution (black solid line) having a standard deviation of 0.47 meV. For comparison, the FWHM of the cavity mode obtained from the emission below threshold is shown in red (~ 7 meV).

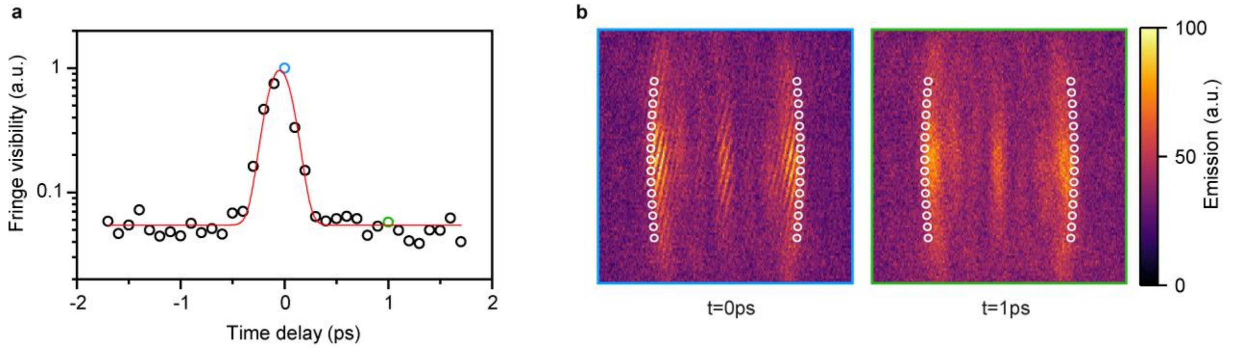

Fig. S8 | Temporal and spatial first-order coherence of the polariton condensate. a, Michelson interference fringe visibility of the polariton condensate emission as a function of time delay between the interferometer arms, as obtained from Fourier transformation of the interferograms. The red line is a Gaussian fit to the data. **b**, Interferometric real-space images at 0 ps and 1 ps time delay, corresponding to the blue and green data points in **a**, respectively. The high fringe contrast observed at the edges originates from out-of-plane scattering by the HCG mirrors. The features in the center of the cavity do not correspond to the internal cavity field distribution but result from imaging artifacts and interference effects, e.g. from downwards scattered light that is partially backreflected by buried the SiO_2/Si interface.

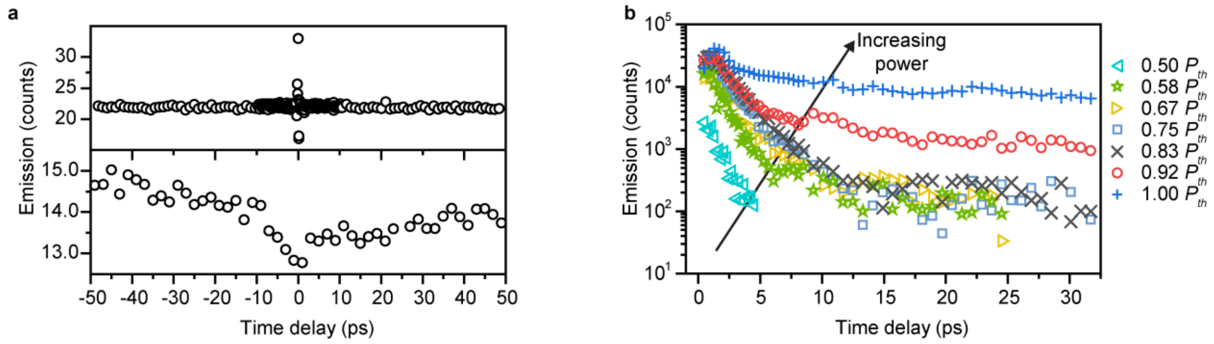

Fig. S9 | Time delay calibration and polariton condensation dynamics. a, The top panel shows the intensity versus time delay for the reflections of two overlapping excitation beams (~150 fs duration), where interference is observed around 0 ps, that is then used as zero calibration point for the dynamical measurements. The bottom panel shows the photoluminescence intensity versus time delay between two overlapping excitation beams for an MeLPPP film without cavity, where the intensity shows a minimum around 0 ps due to nonlinear quenching from exciton annihilation. Notably, the quenching reduces with a ~22 ps time constant, in line with the exciton lifetime for this material. **b**, Integrated intensity of emission spectra of the condensate resulting from the excitation of two spatially overlapping beams on a single HCG cavity as function of time delay for different excitation power (same fluence is used for both beams).

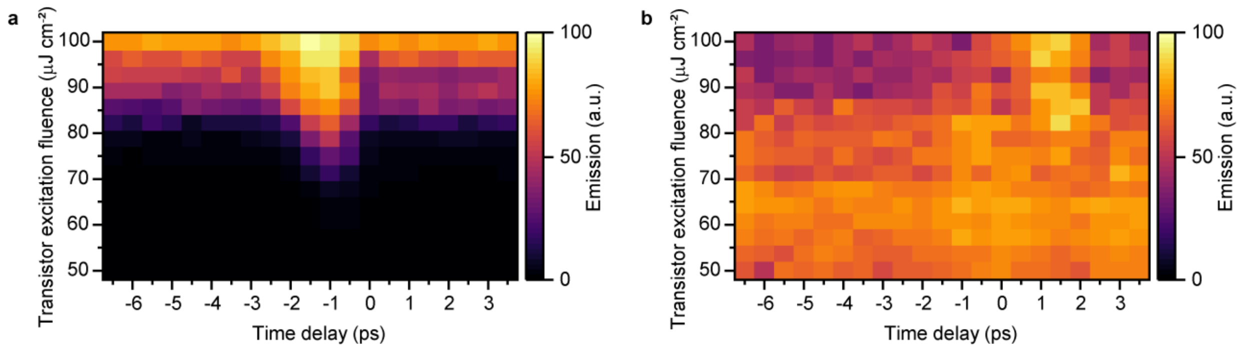

Fig. S10 | Reversing the roles of seed and transistor cavity. a, Real-space emission intensity integrated over the transistor cavity area in the emission image versus transistor excitation fluence and time delay between seed (P_{seed} = 1.5 P_{th}) and transistor excitation, showing a maximum around -1 ps time delay. This analysis of the image intensities yields to almost identical results as the spectral analysis of Fig. 3c in the main manuscript but provides the possibility to analyze simultaneously different structures. **b**, Same experiment as **a**, but here the emission intensity is integrated over the seed cavity area, showing a maximum near +1 ps time delay.

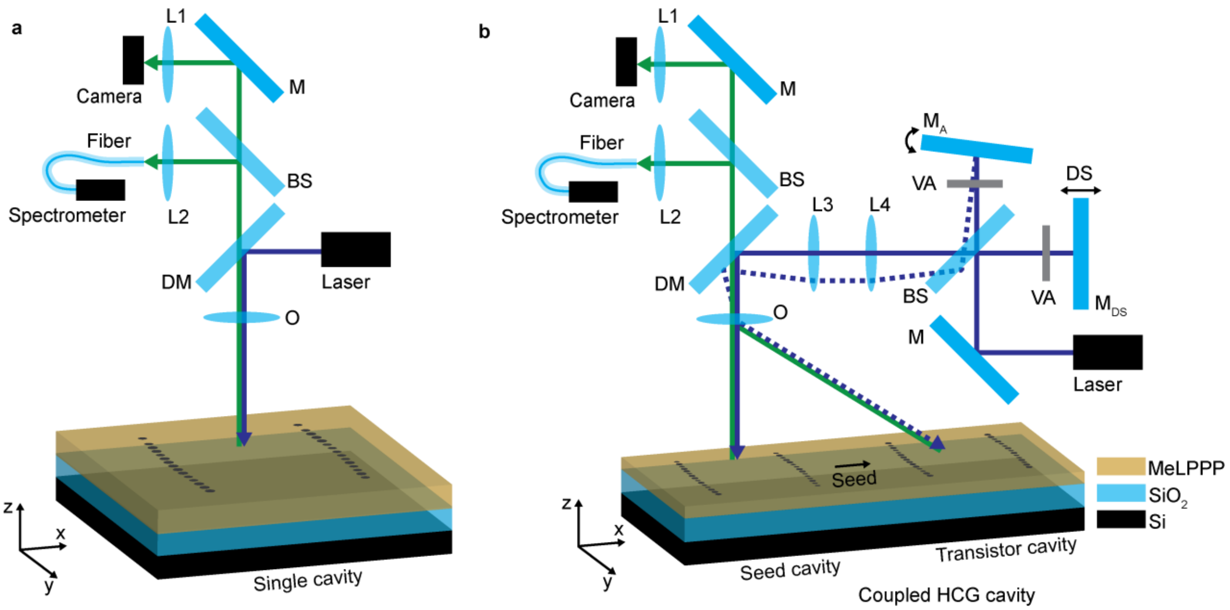

Fig. S11 | Schematic of the measurement setup. a, Configuration used when one HCG device is excited with a single beam. **b**, Configuration used in the measurements involving multiple excitation beams for one or two (dashed lines) HCG devices. M = mirror, L = lens, BS = non-polarizing beam splitter, DM = dichroic mirror, VA = variable attenuator, DS = delay stage, O = objective.

Definition and Extraction of Key Performance Metrics

a. Cavity quality factor

The Quality factor Q is a dimensionless parameter that characterizes the spectral selectivity of a resonance in an optical cavity and is defined as $Q = \frac{E_0}{\Delta E}$, where E_0 is the resonance photon energy, and ΔE is the spectral full width at half maximum (FWHM) of the resonance mode[1]. In our system, Q is extracted from photoluminescence spectra recorded at excitation fluences below threshold, where the cavity response remains linear. From these measurements, we obtain a FWHM of approximately 6 meV, centered at a resonance energy of 2.522 eV, resulting in a Q-factor of ~420, consistent with 3D FDTD calculations.

b. Threshold of polariton condensation

The polariton condensation threshold fluence P_{th} is defined as the excitation fluence at which the emission transitions from the linear photoluminescence to the nonlinear stimulated regime characteristic of polariton condensation. It is identified from the inflection point of the light-in light-out curve, where the emission intensity exhibits a nonlinear increase. As shown in Fig. 2a, we quantify P_{th} from the crossing point of power-law fits below and above the threshold. Apart from the intensity increase, we observe further accompanying spectral signatures that are characteristic of the polariton condensate transition:

- linewidth narrowing of the emission peak,
- continuous blue-shift in emission energy with increasing fluence,
- change in peak shape from Lorentzian (below threshold) to Gaussian (above threshold).

These features are consistent with polariton condensation criteria[2].

c. Amplification

The amplification quantifies how much the seed signal is increased by transistor. Thus, it is obtained from the ratio between the emission intensity $I_{\text{transistor}}$ of the transistor when the seed is present and the transistor pumped (seed and transistor pumped at the optimal delay, i.e. "transistor ON") versus when the seed cavity is excited alone ("transistor OFF"), at the same seed fluence:

$$\text{Amplification} = \frac{I_{\text{transistor}}(\text{"transistor ON"})}{I_{\text{transistor}}(\text{"transistor OFF"})}$$

d. Extinction Ratio

The extinction ratio characterizes the on/off contrast of the transistor. Thus, it is obtained from the ratio between the emission intensity $I_{\text{transistor}}$ of the transistor when the seed is present at optimal delay (Δt = -1 ps), i.e. the transistor input gets a "high" signal, and when the seed pulse arrives with incorrect timing (for example, Δt = +1 ps), i.e. the transistor input effectively gets a "low" signal:

$$\text{Extinction Ratio} = \frac{I_{\text{transistor}}(\Delta t = -1\text{ ps})}{I_{\text{transistor}}(\Delta t = +1\text{ ps})}$$

e. Coupling Efficiency

The coupling efficiency η quantifies what fraction of the optical emission from the seed cavity is injected into the transistor cavity mode. It is estimated from a real-space image taken in the experimental configuration that has the seed cavity excited above threshold and the transistor cavity unpumped such that any detected light from the transistor cavity results solely from scattered seed light. The efficiency is therefore calculated by the ratio of the measured scattered intensity from the far (output) and near (input) HCG mirrors of the transistor cavity:

$$\eta = \frac{I_{\text{transistor,out}}}{I_{\text{transistor,in}}}$$

According to this definition, a high ratio indicates effective coupling. Indeed, if the coupling efficiency were zero, all seed light would be either reflected or scattered at the input side of the transistor cavity, and no significant signal would be observed from the output mirror. The fact that the scattered intensities from both mirrors are comparable supports the conclusion that a large fraction of the seed emission enters and propagates through the transistor cavity mode. While this method does not directly measure the intra-cavity field, it provides a reliable lower-bound estimate for the coupling efficiency based on accessible optical signatures.

f. Switching energy

The switching energy represents the minimum energy required from the seed pulse to trigger polariton condensation in the transistor cavity when this is under sub-threshold excitation. It is estimated by converting the measured integrated intensity (camera counts) of the light scattered out-of-plane from the transistor into the actual energy reaching the cavity, accounting for collection efficiency, optical losses, and detector response. The experimental configuration is such that the seed cavity is excited above threshold and the transistor cavity is unpumped such that any detected emission from the transistor cavity results solely from in-plane propagation of the seeding photons.

$$N_{\text{photons}} = \frac{\left(\frac{Counts}{Shots} \cdot G\right)}{Q_{eff} \cdot BS \cdot (1 - Loss_{Len}) \cdot (1 - Loss_{Obj}) \cdot Vert_{Coll} \cdot S_{up}} \approx 25725$$

Where:

- Counts $= 32144$, integrated real-space counts on the transistor;
- Shots $= 1000$, number of excitation pulses per exposure time;
- $G = 1.4$, camera gain;
- $Q_{\text{eff}} = 50\%$,: quantum efficiency of the detector;
- $BS = 50\%$, beam splitter ratio (for splitting the signal between spectrometer and camera);
- $\text{Loss}_{\text{Len}} = 0.03 \times 4 = 12\%$, losses from four lenses and mirrors;
- $\text{Loss}_{\text{Obj}} = 10\%$, transmission loss from the objective;
- $\text{Vert}_{\text{Coll}} = 1 - \cos(\arcsin(NA)) = 28.5\%$, vertical collection efficiency given $NA = 0.7$ of the objective and assuming isotropic scattering;
- $S_{up} = \sim 0.031$, fraction of light-scattered upwards by the HCG, as detailed more below;

With a photon energy of $E_{\text{photon}} = 2.52$ eV $= 4.04 \times 10^{-19}$ J, we can estimate the total switching energy as:

$$E_{switch} = N_{photons} \cdot E_{photon} \approx 10.4 \text{ fJ}$$

In order to estimate the upwards, towards the detector scattered light $S_{up} = \sim 0.031$ we consider the scattering and absorption processes occurring in the HCGs, as illustrated in Fig. S12.

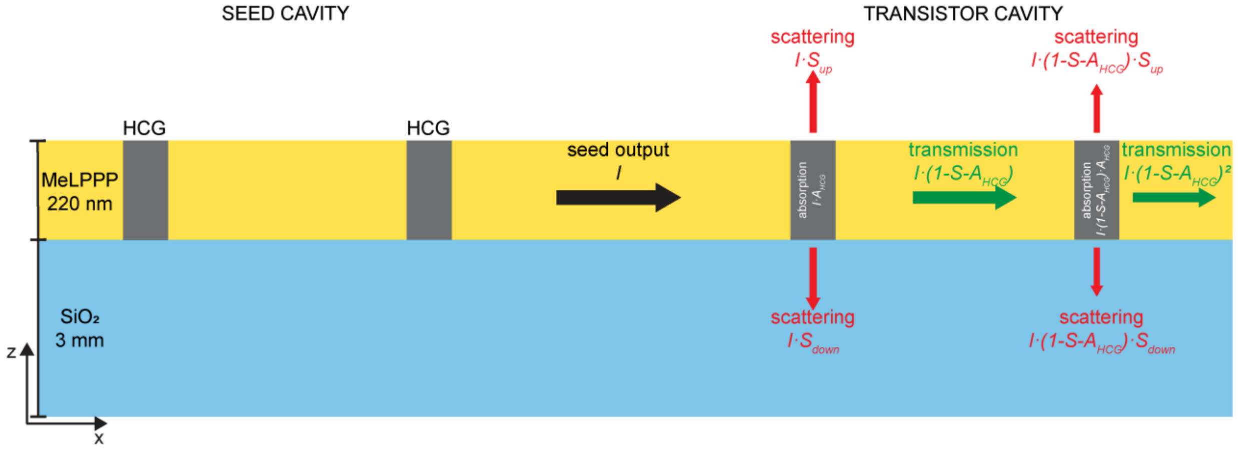

Fig. S12 | Schematic of seed-to-transistor coupling and mirror losses. Cross-section conceptual diagram (xz plane) showing the HCG mirrors (gray) of the seed cavity on the left and the transistor cavity on the right, with the polymer MeLPPP (yellow) providing vertical guiding of the light. The seed cavity emits an in-plane optical signal (black arrow), which propagates toward the transistor cavity. Upon reaching the input HCG mirror of the transistor, the incoming light intensity I undergoes partial scattering (red arrows), absorption (white, inside silicon HCG), and transmission (green rightward arrows). The scattering fractions S_{up} and S_{down}, absorption A_{HCG}, and transmission $(1 - S - A_{\mathrm{HCG}})$ (with $S = S_{up} + S_{down}$) define the energy flow at each mirror, as used to estimate the fraction of light scattered upwards from the transistor cavity to be detected by the camera for the switching energy calculation.

In our experiment, the coupling efficiency η to provide a bound for the total energy lost at each HCG through absorption and scattering, as

$$S_{up} + S_{down} + A_{HCG} = 1 - \eta = 16\%$$

In order to estimate the absorbed fraction A_{HCG}, we consider that the HCG mirrors are composed of $d = 105$ nm diameter silicon pillars with $p = 165$ nm pitch, corresponding to an effective fill factor of

$$f = \frac{\pi(d/2)^2}{d \cdot p} = 47.5\%$$

To estimate the effective absorption per pass through the HCG, with the silicon absorption coefficient ($\alpha = 19536\ \mathrm{cm}^{-1}$ at 2.52 eV photon energy)[3], we can write:

$$A_{HCG} = f(1 - e^{-\alpha d}) = 8.4\%$$

From the refractive indices above ($n_{\mathrm{air}} = 1$) and below ($n_{\mathrm{SiO2}} = 1.46$) the HCG, we assume a slightly preferential downwards scattering, i.e. $S_{\mathrm{down}} = 1.5\ S_{\mathrm{up}}$. Thereby we obtain for S_{up}:

$$\mathrm{S}_{up} = \frac{1 - \eta - A_{HCG}}{2.46} = 1 - \eta = 3.1\%.$$

Table S1 | Benchmark of all-optical logic and switching devices.

Comparison of reported all-optical switches and logic gates, including our integrated polariton-based all-optical transistor. '—' denotes that data is not available.

Ref.	System	Switching time (ps)	Gain (dB)	Switching Energy	Footprint (μm²)	Integration Capability
This work	Organic microcavity	~1	~18	~10 fJ	4	High
Zasedatelev *et al.* 2019[4]	Organic microcavity	~1	~35	~80 fJ	~500	Low: external re-routing, large area
Ballarini *et al.* 2013[5]	GaAs microcavity	~10	~13	~1.3 fJ	~1000	Low: cryogenic, large area
Gao *et al.* 2012[6]	Organic microcavity	~18	—	~100 fJ	~1000	Low: slow, high loss, cryogenic, large area,
Kumar *et al.* 2006[7]	SOA	~25	—	15 pJ	module	Medium: mature tech, but large & power
Dong *et al.* 2008[8]	SOA	25	12	12 fJ	module	Medium
Lai *et al.* 2008[9]	Optical fiber	~5	—	—	>10^6	Low: large-scale
Wang *et al.* 2011[10]	Optical fiber	100	—	—	>10^6	Very Low
Liang *et al.* 2006[11]	Si waveguide	12.5	—	~8 pJ	~1000	High: CMOS-compatible
Uddin *et al.* 2009[12]	FP laser diode	25	—	30 fJ	—	Medium
Feng *et al.* 2013[13]	InGaAs/AlAsS quantum wells	2.4	—	5.6 pJ	~1000	Medium
Nozaki *et al.* 2010[14]	InGaAsP photonic-crystal	~10	—	0.66 fJ	10	Medium (III–V)
Almeida *et al.* 2004[15]	Si ring resonators	~450	—	25 pJ	~500	High (CMOS)
Ono/ *et al.* 2020[16]	Graphene plasmonic	0.26	—	35 fJ	~150	Low
Guo *et al.* 2022[17]	Lithium niobate	0.046	—	80 fJ	>10^5	Low
Li et al. 2024[18]	ZnO microwire	0.08	—	<100 pJ	—	Low

References

1. Saleh, B. E. A. & Teich, M. C. *Fundamentals of Photonics*. (Wiley-Interscience, Hoboken, N.J, 2007).
2. Deng, H., Haug, H. & Yamamoto, Y. Exciton-polariton Bose-Einstein condensation. *Rev. Mod. Phys.* **82**, 1489–1537 (2010).
3. Schinke, C. *et al.* Uncertainty analysis for the coefficient of band-to-band absorption of crystalline silicon. *AIP Adv.* **5**, 067168 (2015).
4. Zasedatelev, A. V. *et al.* A room-temperature organic polariton transistor. *Nat. Photonics* **13**, 378–383 (2019).
5. Ballarini, D. *et al.* All-optical polariton transistor. *Nat. Commun.* **4**, 1778 (2013).
6. Gao, T. *et al.* Polariton condensate transistor switch. *Phys. Rev. B* **85**, 235102 (2012).
7. Kumar, S. & Willner, A. E. Simultaneous four-wave mixing and cross-gain modulation for implementing an all-optical XNOR logic gate using a single SOA. *Opt. Express* **14**, 5092–5097 (2006).
8. Dong, J., Zhang, X., Xu, J. & Huang, D. 40 Gb/s all-optical logic NOR and OR gates using a semiconductor optical amplifier: Experimental demonstration and theoretical analysis. *Opt. Commun.* **281**, 1710–1715 (2008).
9. Lai, D. M. F., Kwok, C. H. & Wong, K. K.-Y. All-optical picoseconds logic gates based on a fiber optical parametric amplifier. *Opt. Express* **16**, 18362–18370 (2008).
10. Wang, W. *et al.* Experimental Research on 10 Gb/s All-Optical Logic Gates with Return-to-zero Data in High Nonlinear Fiber. in *Optoelectronic Materials and Devices (2011), paper 830829* 830829 (Optica Publishing Group, 2011). doi:10.1364/ACP.2011.830829.
11. Liang, T. K. *et al.* High speed logic gate using two-photon absorption in silicon waveguides. *Opt. Commun.* **265**, 171–174 (2006).
12. Rakib Uddin, M., Lim, J. S., Jeong, Y. D. & Won, Y. H. All-Optical Digital Logic Gates Using Single-Mode Fabry–PÉrot Laser Diode. *IEEE Photonics Technol. Lett.* **21**, 1468–1470 (2009).
13. Feng, J. *et al.* Band edge tailoring of InGaAs/AlAsSb coupled double quantum wells for a monolithically integrated all-optical switch. *Opt. Express* **21**, 15840–15846 (2013).
14. Nozaki, K. *et al.* Sub-femtojoule all-optical switching using a photonic-crystal nanocavity. *Nat. Photonics* **4**, 477–483 (2010).
15. Almeida, V. R., Barrios, C. A., Panepucci, R. R. & Lipson, M. All-optical control of light on a silicon chip. *Nature* **431**, 1081–1084 (2004).
16. Ono, M. *et al.* Ultrafast and energy-efficient all-optical switching with graphene-loaded deep-subwavelength plasmonic waveguides. *Nat. Photonics* **14**, 37–43 (2020).
17. Guo, Q. *et al.* Femtojoule femtosecond all-optical switching in lithium niobate nanophotonics. *Nat. Photonics* **16**, 625–631 (2022).
18. Li, H. *et al.* All-optical temporal logic gates in localized exciton polaritons. *Nat. Photonics* **18**, 864–869 (2024).
